# Supplementary material for: Development of a short questionnaire based on the Practice Environment Scale-Nursing Work Index in primary health care
Source: PeerJ. 2019 Jul 24;7:e7369. doi: 10.7717/peerj.7369 (PMC6660900; doi:10.7717/peerj.7369)
Supplement: Supplemental Information 1 — Questionnaire used in the study. [file peerj-07-7369-s001.docx]

**A.1: Appendice 1.**

**Spanish Version (31 items), Practice Environment Scale of the Nursing Work Index (PES-NWI).**

(Permission was obtained to use the questionnaire).

De Pedro-Gómez, J. et al, 2012.

| DIMENSIÓN | Nº DE ÍTEM | | DESCRIPCIÓN ÍTEM | |  | |  |  |  |
| --- | --- | --- | --- | --- | --- | --- | --- | --- | --- |
|  |  | | **DIMENSIÓN 1: PARTICIPACIÓN EN LOS ASUNTOS DEL CENTRO.** | |  | |  |  |  |
| 1 | 1 | | | Las enfermeras de plantilla están formalmente involucradas en la gestión interna del centro (juntas, órganos de decisión.) | 1 | | 2 | 3 | 4 |
| 1 | 2 | | | Las enfermeras del centro tienen oportunidades para participar en las decisiones que afectan a las distintas políticas que desarrolla el mismo. | 1 | | 2 | 3 | 4 |
| 1 | 3 | | | Existen muchas oportunidades para el desarrollo profesional del personal de Enfermería. | 1 | | 2 | 3 | 4 |
| 1 | 4 | | | La Dirección escucha y da respuesta a los asuntos de sus enfermeras. | 1 | | 2 | 3 | 4 |
| 1 | 5 | | | El/la Director/a de Enfermería es accesible y fácilmente “visible”. | 1 | | 2 | 3 | 4 |
| 1 | 6 | | | Se puede desarrollar una carrera profesional o hay oportunidades de ascenso en la carrera clínica. | 1 | | 2 | 3 | 4 |
| 1 | 7 | | | Los gestores enfermeros consultan con las enfermeras los problemas y modos de hacer del día a día. | 1 | | 2 | 3 | 4 |
| 1 | 8 | | | Las enfermeras de plantilla tienen oportunidades para participar en las comisiones del centro, tales como la comisión de investigación, de ética, de infecciones… | 1 | | 2 | 3 | 4 |
| 1 | 9 | | | Los directivos enfermeros están al mismo nivel en poder y autoridad que el resto de directivos del centro. | 1 | | 2 | 3 | 4 |
|  |  | | | **DIMENSIÓN 2: FUNDAMENTO PARA LA CALIDAD DEL CUIDADO.** |  | |  |  |  |
| 2 | 10 | | | Se usan los diagnósticos enfermeros. | 1 | | 2 | 3 | 4 |
| 2 | 11 | | | Hay un programa activo de garantía y mejora calidad. | 1 | | 2 | 3 | 4 |
| 2 | 12 | | | Existe un programa de acogida y tutelaje de enfermeras de nuevo ingreso. | 1 | | 2 | 3 | 4 |
| 2 | 13 | | | Los cuidados de las enfermeras están basados en un modelo enfermero, más que en un modelo biomédico. | 1 | | 2 | 3 | 4 |
| 2 | 14 | | | La asignación de pacientes a cada enfermera existente favorece la continuidad de los cuidados (p. ej.: la misma enfermera cuida al paciente a lo largo del tiempo) | 1 | | 2 | 3 | 4 |
| 2 | 15 | | | Hay una filosofía común de Enfermería, bien definida, que impregna el entorno en el que se cuida a los pacientes | 1 | | 2 | 3 | 4 |
| 2 | 16 | | | Existe un plan de cuidados escrito y actualizado para cada paciente. | 1 | | 2 | 3 | 4 |
| 2 | 17 | | | Los gestores del centro se preocupan de que los enfermeros proporcionen cuidados de alta calidad. | 1 | | 2 | 3 | 4 |
| 2 | 18 | | | Se desarrollan programas de formación continuada para las enfermeras. | 1 | | 2 | 3 | 4 |
| 2 | 19 | | | Las enfermeras del centro tienen una competencia clínica adecuada. | 1 | | 2 | 3 | 4 |
|  |  | | | **DIMENSIÓN 3: APOYO DE LA COORDINACIÓN** |  | |  |  |  |
| 3 | 20 | | | La supervisora/coordinadora es una buena gestora y líder. | 1 | | 2 | 3 | 4 |
| 3 | 21 | | | La supervisora/coordinadora respalda a la plantilla en sus decisiones, incluso si el conflicto es con personal médico | 1 | | 2 | 3 | 4 |
| 3 | 22 | | | La supervisora/coordinadora utiliza los errores como oportunidades de aprendizaje y mejora, no como crítica. | 1 | | 2 | 3 | 4 |
| 3 | 23 | | | La supervisora/coordinadora es comprensiva y asesora y da apoyo a las enfermeras. | 1 | | 2 | 3 | 4 |
| 3 | 24 | | | Se reconoce y elogia el trabajo bien hecho. | 1 | | 2 | 3 | 4 |
|  |  | | | **DIMENSIÓN 4: RECURSOS HUMANOS** |  | |  |  |  |
| 4 | 25 | | | Hay suficiente plantilla de empleados para realizar adecuadamente el trabajo. | 1 | | 2 | 3 | 4 |
| 4 | 26 | | | Hay suficiente número de enfermeras diplomadas para proveer cuidados de calidad. | 1 | | 2 | 3 | 4 |
| 4 | 27 | | | Los servicios de apoyo (celadores, administrativos…) son adecuados y facilitan estar más tiempo con los pacientes. | 1 | | 2 | 3 | 4 |
| 4 | 28 | | | Hay tiempo suficiente y oportunidad para discutir los problemas de cuidados con las otras enfermeras. | 1 | | 2 | 3 | 4 |
|  | |  | | **DIMENSIÓN 5**  **RELACIONES MÉDICOS / ENFERMERAS.** | |  |  |  |  |
| 5 | 29 | | | Se realiza mucho trabajo en equipo entre médicos y enfermeras. | 1 | | 2 | 3 | 4 |
| 5 | 30 | | | Entre los médicos y las enfermeras se dan buenas relaciones de trabajo. | 1 | | 2 | 3 | 4 |
| 5 | 31 | | | La práctica entre enfermeras y médicos está basada en una colaboración apropiada. | 1 | | 2 | 3 | 4 |
